# Radiomics and ischemic stroke research: bibliometric insights and visual trends (2004–2024)

**DOI:** 10.3389/fneur.2025.1606388

**Published:** 2025-08-28

**Authors:** Jiacheng Zhang, Hainan Zhu, Hengzhen Wu, Huabao Xie, Dingyi Lin, Lielie Zhu

**Affiliations:** ^1^Department of Rehabilitation, Wenzhou Ouhai District Third People’s Hospital, Wenzhou, China; ^2^Department of Rehabilitation, Taishun County Hospital of Traditional Chinese Medicine (Taishun County TCM Medical Community), Wenzhou, China; ^3^Department of Rehabilitation, Wenzhou TCM Hospital of Zhejiang Chinese Medical University, Wenzhou, China

**Keywords:** radiomics, ischemic stroke, visualization, bibliometrics, CiteSpace, VOSviewer

## Abstract

**Background:**

Ischemic stroke is a leading global cause of death and disability, presenting significant challenges in diagnosis, treatment, and prognosis. Radiomics, an emerging interdisciplinary methodology, employs machine learning to extract high-dimensional features from medical imaging and has demonstrated superior predictive performance in ischemic stroke research. However, the rapidly accumulating publications lack systematic bibliometric synthesis. We therefore conducted a visual bibliometric analysis to map research evolution and emerging trends.

**Methods:**

This study conducted a bibliometric and visual analysis of ischemic stroke radiomics research from 2004 to 2024 using tools like CiteSpace and VOSviewer. The analysis explored publication trends, research hotspots, and technological advancements, identifying collaborations and key advancements in the field.

**Results:**

Radiomics research in ischemic stroke has grown exponentially since its inception in 2014, with China and the United States emerging as major contributors. The primary focus has been on AIS, utilizing advanced imaging techniques such as computed tomography (CT) and magnetic resonance imaging (MRI). Machine learning models, particularly deep learning architectures, are being widely applied for lesion segmentation, risk assessment, and functional prognosis prediction. Despite rapid advancements, challenges persist in standardizing imaging protocols, enhancing interdisciplinary collaborations, and ensuring clinical translation.

**Conclusion:**

Radiomics is transforming ischemic stroke research by enabling detailed imaging analyses and facilitating data-driven clinical decision-making. Future endeavors should prioritize addressing standardization issues, expanding multicenter collaborations, and developing interpretable models that integrate radiomics with clinical and molecular biomarkers. Such efforts will accelerate the translation of radiomics into routine ischemic stroke care and improve patient outcomes.

## 1 Introduction

Stroke, as the second leading global cause of death and third leading cause of disability, imposes a lifelong health risk affecting approximately one-quarter of the population (1). In 2021, the number of people suffering from stroke worldwide reached 57.3 million, with 5.03 million deaths (2). Ischemic stroke is caused by blockage of the cerebral arteries and accounts for approximately 87% of all stroke cases (3). A 2024 study predicts that by 2050, global DALYs due to stroke will rise from the third position in 2022 to the second, surpassed only by ischemic heart disease (4). Furthermore, a US-based study forecasts that the stroke rate among Americans will increase from 3.9% in 2020 to 6.4% by 2050 (5). Currently, the diagnosis and treatment of stroke encounter various challenges, including constraints in the early diagnosis and treatment window for acute ischemic stroke (AIS) patients and difficulties in accurately evaluating long-term outcomes. Consequently, advancing technological methods to enhance ischemic stroke diagnosis, treatment, and prognosis management holds significant importance.

Radiomics, an emerging multidisciplinary technique, covers the extraction of high-throughput quantitative features from medical images combined with machine learning algorithms to deeply explore the underlying biological information within the imaging data (6, 7). In recent years, the application of radiomics in stroke research has garnered increasing attention, demonstrating significant potential in disease diagnosis, prognosis prediction, and personalized treatment. For instance, radiomics features based on magnetic resonance imaging (MRI) have been utilized to predict functional outcomes in patients with acute ischemic stroke, achieving an area under the ROC curve (AUC) of 0.92 for the prediction model (8). Additionally, radiomics can be employed to evaluate hematoma volume and growth risk in patients with cerebral hemorrhage (3).

With the rapid advancement of ischemic stroke and radiomics research, the literature in these fields has experienced exponential growth. Comprehensively and systematically organizing existing research outcomes, as well as identifying key research hotspots and technological trends, has become a crucial task for the academic community. Bibliometric analysis methods, such as co-citation analysis and keyword clustering, have proven to be effective tools in revealing the current state and cutting-edge trends of research (9). However, bibliometric studies specifically targeting the field of ischemic stroke radiomics are currently scarce, lacking systematic and visual analysis.

Therefore, this study focuses on ischemic stroke radiomics-related literature from 2004 to 2024, employing bibliometric and visual analysis methods with the use of CiteSpace and VOSviewer software. The aim is to comprehensively explore the research hotspots and development trends in this domain, providing guidance for future basic research and clinical applications, and offering significant reference value to relevant researchers.

## 2 Materials and methods

### 2.1 Searching strategy

The data analysis in this article is based on the Web of Science Core Collection (WoSCC), a database published by Clarivate Analytics. To cover as many relevant research papers as possible, we constructed a search strategy using commonly used terms in scientific literature. The terms “ischemic stroke,” “radiomics,” and their synonyms were sourced from the PubMed Medical Subject Headings (MeSH) database. The search was completed on February 8, 2025, and the search queries and details are provided in Supplementary Table S1.

### 2.2 Inclusion and exclusion criteria

#### 2.2.1 Inclusion criteria

(1) The study population must consist of patients diagnosed with any type of ischemic stroke. (2) The research should utilize radiomics-related methodologies. (3) Only clinical studies conducted on humans, published in English, and presented in article format were eligible. (4) Studies must have been published between January 1, 2004, and December 31, 2024. (5) Eligible article types were limited to original research articles or review papers.

#### 2.2.2 Exclusion criteria

(1) Articles not focusing on the application of radiomics in stroke were excluded. (2) Unpublished works or those with “early access” status were not considered. (3) Studies written in languages other than English were excluded. Further details on the study selection process can be found in Supplementary Figure S1.

### 2.3 Data collection

The process of data curation and screening for this study was conducted as follows: (1) Two independent researchers from the team assessed the articles to determine their relevance to the study’s focus, excluding those that did not satisfy the eligibility criteria. Any disagreements were resolved through discussion. (2) Affiliation details and country names were revised and standardized to minimize potential biases in the results. (3) Keywords were also standardized to resolve inconsistencies caused by differences in word forms, such as plurals and singulars, which might otherwise lead to redundant entries in the keyword co-occurrence analysis. For instance, “Peoples R China” and “Taiwan” were standardized as “China,” while “England,” “North Ireland,” “Wales,” and “Scotland” were merged into “United Kingdom.”

In total, 213 records were initially retrieved. Following an independent review conducted by two researchers, 204 records were finalized for inclusion in the study. The dataset was exported as a plain text file containing details such as publication year, title, abstract, author names, institutions, journal names, and keywords.

### 2.4 Data analysis

The bibliometric data retrieved from the database was stored in files for subsequent analysis. Temporal publication trends were analyzed using Microsoft Excel. For constructing visual network maps, VOSviewer (1.6.18) was employed, a tool that utilizes a probabilistic approach to data standardization. This enabled the creation of detailed visualizations showcasing publishing countries, contributing institutions, influential authors, publishing journals, and key cited references. In these visualizations, the size of each node represented its connection degree, connection strength, and frequency of occurrence, while the thickness of connecting lines indicated the level of collaboration between nodes. Node colors distinguished different clusters within the network. This approach provided a multidimensional perspective on the research landscape in the field. Additionally, Microsoft Excel 2016 and Scimago Graphica were employed for data integration and other visualization tasks.

To create knowledge mapping visualizations, CiteSpace (V6.3.R1) software was used. The analysis settings included time slicing set to 1, covering the years 2004 to 2024 with annual intervals. A time-based similarity algorithm was applied to generate two key visualizations: timeline plots and keyword burst analyses. Timeline plots, displayed as cluster diagrams, depicted the evolution of keyword-related clusters over time, shedding light on the chronological development of the research field. Keyword burst analysis played a crucial role in detecting emerging trends by identifying abrupt increases in the usage frequency of specific terms. Together, these analytical methods facilitated an in-depth examination of both historical changes in research focus and emerging directions, offering a detailed understanding of the field’s progression and future potential.

## 3 Results

### 3.1 Analysis of annual publications

**Figure 1:**
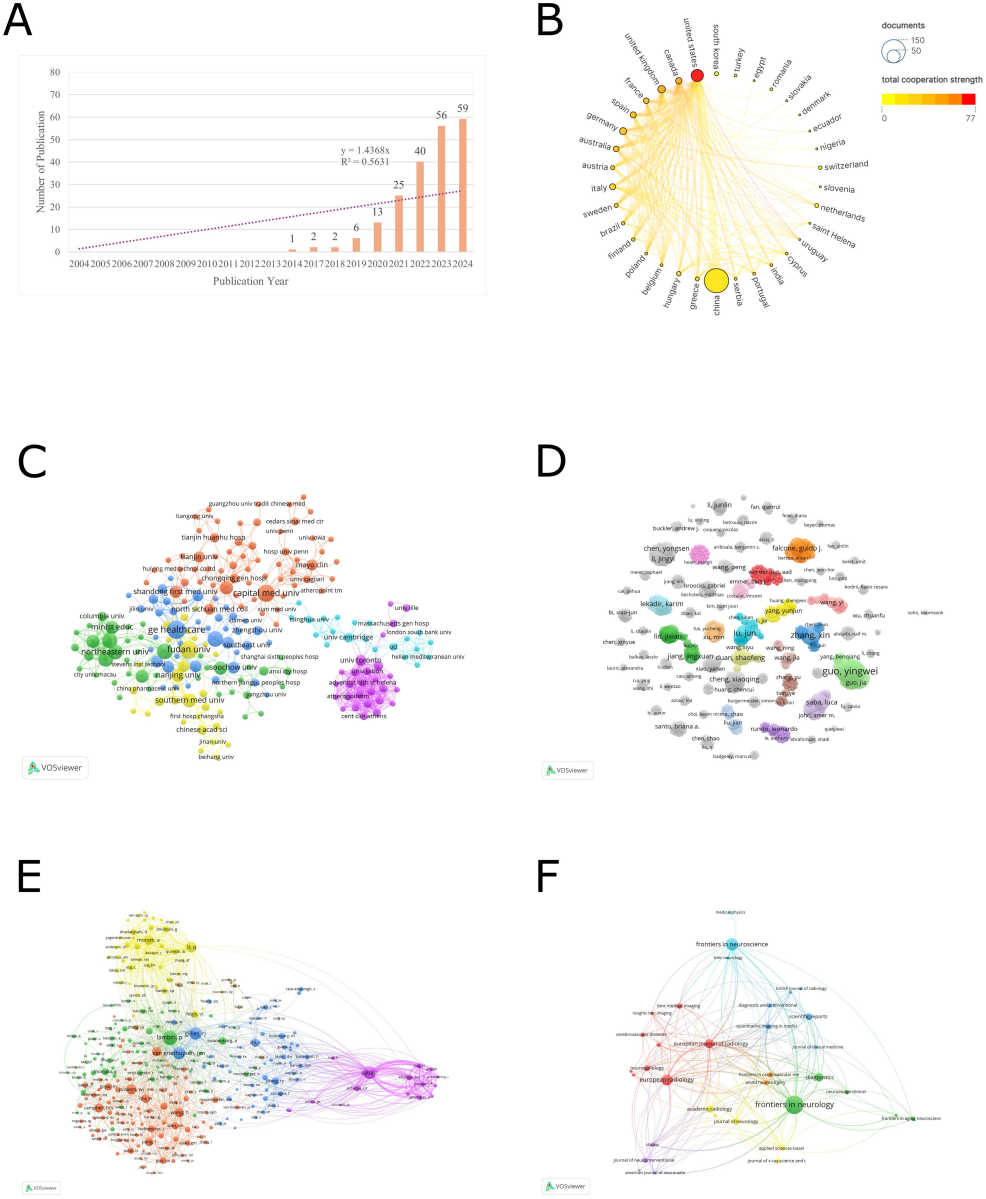
**(A)** The trend in the number of radiomics studies published in the field of stroke from 2004 to 2024. **(B)** Countries cooperation analysis. **(C)** Organizations cooperation analysis. **(D)** Authors cooperation analysis. **(E)** Co-cited author analysis. **(F)** Journal analysis.

According to Figure 1A, the application of radiomics in the field of ischemic stroke emerged with the first study in 2014. Since then, it has gradually garnered the attention of global researchers. Notably, after 2021, there was a significant increase in the number of publications. In 2024, 59 papers were published in just 1 year, with a total of 204 papers published by the end of 2024. The trend line formula is *y* = 1.4368*x*, with an *R*^2^ value of 0.5631, indicating a linear growth trend.

### 3.2 Analysis of countries

Based on the analysis conducted by VOSviewer, co-authorship was selected for the type of analysis and countries were selected for the unit of analysis, the threshold of the minimum number of publications in a country was selected as 1, a total of 34 countries have participated in publishing radiomics-related articles in the field of ischemic stroke. Table 1 presents the ranking of the top 10 countries by the number of publications. In terms of publication volume, only China has exceeded 100 articles, reaching a total of 147 articles, which is significantly ahead of other countries. This is followed by the United States (37), the United Kingdom (14), Germany (12), and Canada (10). Regarding total citations, the top 5 countries are China (1,313), the United States (567), Germany (223), Australia (202), and the United Kingdom. Figure 1B illustrates the visual network of countries ranked by their total collaboration strength. In this figure, the countries are sorted by collaboration intensity, where colors closer to red indicate higher collaboration strength, and larger nodes represent a higher number of publications. In terms of collaboration intensity, the United States (77) has the closest cross-border collaboration in this field, followed by Canada (53), the United Kingdom (50), France (37), and Spain (35).

**Table 1:** Top 10 countries in number of papers published in the field of radiomics for stroke.

	Countries/Regions	Documents	Citations	Total cooperation strength
1	China	147	1,313	19
2	United States	37	567	77
3	United Kingdom	14	192	50
4	Germany	12	223	34
5	Canada	10	188	53
6	Spain	9	121	35
7	Italy	9	49	28
8	Australia	8	202	34
9	France	8	126	37
10	Hungary	5	36	23

### 3.3 Analysis of organizations

We conducted an institutional collaboration network analysis using VOSviewer software on relevant literature in the field of radiomics in ischemic stroke. Co-authorship was selected for the type of analysis and organizations were selected for the unit of analysis, the threshold of the minimum number of publications in a country was selected as 1. The analysis revealed a total of 425 institutions that have published at least one article in this domain. Table 2 presents the ranking of the top 15 organizations based on productivity. General Electric Healthcare leads the list with 13 publications, followed by Fudan University (12), Capital Medical University (11), Nanjing Medical University (9), and Shanghai Jiao Tong University (9). In terms of total citations, the top five organizations are Capital Medical University (212), General Electric Healthcare (172), Fudan University (154), Wenzhou Medical University (145) - despite Wenzhou Medical University having only 5 publications. Regarding collaboration intensity, the University of Toronto stands out with 46 collaborations, even though it has only 4 publications. This is followed by Tongji University (42), Northeastern University (41), and Shenzhen Technology University (41). It is noteworthy that among the top 15 organizations, all except the first one from the United States are from China.

**Table 2:** Top 15 organizations in number of papers published in the field of radiomics for stroke.

	Organization	Documents	Citations	Total cooperation strength	Country
1	General Electric Healthcare	13	172	32	United States
2	Fudan University	12	154	15	China
3	Capital Medical University	11	212	22	China
4	Nanjing Medical University	9	111	15	China
5	Shanghai Jiao Tong University	9	91	27	China
6	Northeastern University	8	50	41	China
7	Shenzhen Technology University	8	50	41	China
8	Tongji University	8	33	42	China
9	Southern Medical University	7	95	16	China
10	Nanjing University	6	61	18	China
11	Zhejiang University	6	53	11	China
12	Ministry of Education	6	33	32	China
13	Nantong University	6	30	13	China
14	Chongqing Medical University	6	29	22	China
15	Shenzhen University	6	29	33	China

Figure 1C illustrates the visual network of the largest subset of 218 organizations with collaborative relationships (6 clusters). Each node corresponds to an organization, with its size reflecting the number of publications. The connecting lines indicate collaborative relationships between organizations, while their thickness denotes the strength of these collaborations. Distinct colors are used to represent different clusters. Prominent organizations such as General Electric Healthcare, Northeastern University, Fudan University, Capital Medical University, University of Toronto, and University of Cambridge are visible with larger nodes and numerous connections, indicating their high publication output and extensive collaborative efforts.

### 3.4 Analysis of authors and co-cited authors

Using VOSviewer software, we conducted an analysis of publishing authors and co-cited authors to identify their key contributions to the field. Table 3 presents the top 10 authors who have published the most radiomics-related papers in the ischemic stroke field, as well as the top 15 authors who have been co-cited the most in this field (co-citation refers to when articles by two authors are cited by the same paper, counting as one co-citation). In terms of publication volume, all top 10 authors are from China. Kang, Yan (8) and Guo, Yingwei (8) are tied for the first place in publication volume, followed closely by Luo, Yu (7) and Zeng, Xueqiang (7). Regarding co-citations, the top 15 authors mainly come from Belgium, Italy, United States, Netherlands, China, and Australia. Lambin, P. (73) ranks first in the number of citations, followed by Saba, L. (61), Gillies, R. J. (59), and van Griethuysen, J. J. M. (58).

**Table 3:** Top 10 authors in number of papers published and top 15 co-citied authors in the field of radiomics for stroke.

	Author	Documents	Citations	Country		Co-cited author	Co-cited	Country
1	Kang, Yan	8	50	China	1	Lambin, P.	73	Belgium
2	Guo, Yingwei	8	33	China	2	Saba, L.	61	Italy
3	Luo, Yu	7	33	China	3	Gillies, R. J.	59	United States
4	Zeng, Xueqiang	7	33	China	4	van Griethuysen, J. J. M.	58	Netherlands
5	Zhang, Xin	6	77	China	5	Li, Q.	44	China
6	Yang, Yingjian	6	30	China	6	Powers, W. J.	42	United States
7	Zaman, Asim	6	29	China	7	Morotti, A.	38	Italy
8	Cao, Fengqiu	5	30	China	8	Wang, H.	36	China
9	Lu, Jun	5	30	China	9	Chen, Q.	35	China
10	Lu, Jiaxi	5	19	China	10	Campbell, B. C. V.	33	Australia
					11	Qiu, W.	31	China
					12	Shi, Z.	30	China
					13	Jiang, L.	29	China
					14	Zwanenburg, A.	26	Netherlands
					15	Zhang, R. Y.	25	China

Figure 1D displays a network of 911 authors who have published at least one article and been cited at least five times. Each node represents an author, and its size reflects the number of publications. The lines connecting the nodes signify collaborative relationships between authors, with their thickness indicating the intensity of collaboration. Different colors distinguish various clusters. Although large-scale collaborative clusters have not yet formed, small-scale clusters of author collaboration can be observed in the figure.

Figure 1E shows a network of 289 authors with at least five co-citations (six clusters). In the blue cluster, Lambin, P. and Gillies, R. J. are the core, indicating their significant influence in the collaboration network. The purple cluster, centered around Saba, L., forms an independent collaborative group with relatively few connections to other clusters.

### 3.5 Analysis of journals

An analysis of publishing journals was conducted using VOSviewer software to identify journals interested in this field. Citation was selected for the type of analysis and sources were selected for the unit of analysis, the threshold of the minimum number of publications in a journal was selected as 2. Table 4 presents the top 10 journals with the highest publication volume in this domain. Among them, *Frontiers in Neurology* stands out as the journal with the most publications, totaling 26 articles, followed by *Frontiers in Neuroscience* with 14 articles. In terms of total citations, *European Radiology* ranks first with a total of 256 citations for its articles in this field, closely followed by *Diagnostics* with 153 citations. Regarding impact factors, the range spans from a minimum of 2.4 to a maximum of 4.8. From the perspective of JCR partitions, the majority are concentrated in Q1 and Q2. Figure 1F illustrates a visual network of 35 journals (forming 7 clusters) among the 36 journals that have published more than 2 articles and share citation relationships.

**Table 4:** Top 10 journals in number of papers published in the field of radiomics for stroke.

	Source	Documents	Citations	Total link strength	IF (2023)
1	Frontiers in Neurology	26	110	96	2.7/Q2
2	Frontiers in Neuroscience	14	145	56	3.2/Q2
3	European Radiology	12	256	61	4.7/Q1
4	European Journal of Radiology	9	78	76	3.2/Q1
5	Diagnostics	8	153	36	3.0/Q1
6	Scientific Reports	6	55	21	3.8/Q1
7	Academic Radiology	6	50	19	3.8/Q1
8	Journal of Neurology	5	112	31	4.8/Q1
9	Neuroradiology	5	45	23	2.4/Q2
10	Journal of Neurointerventional Surgery	4	125	**18**	4.5/Q1

### 3.6 Analysis of highly cited and bursting references

**Figure 2:**
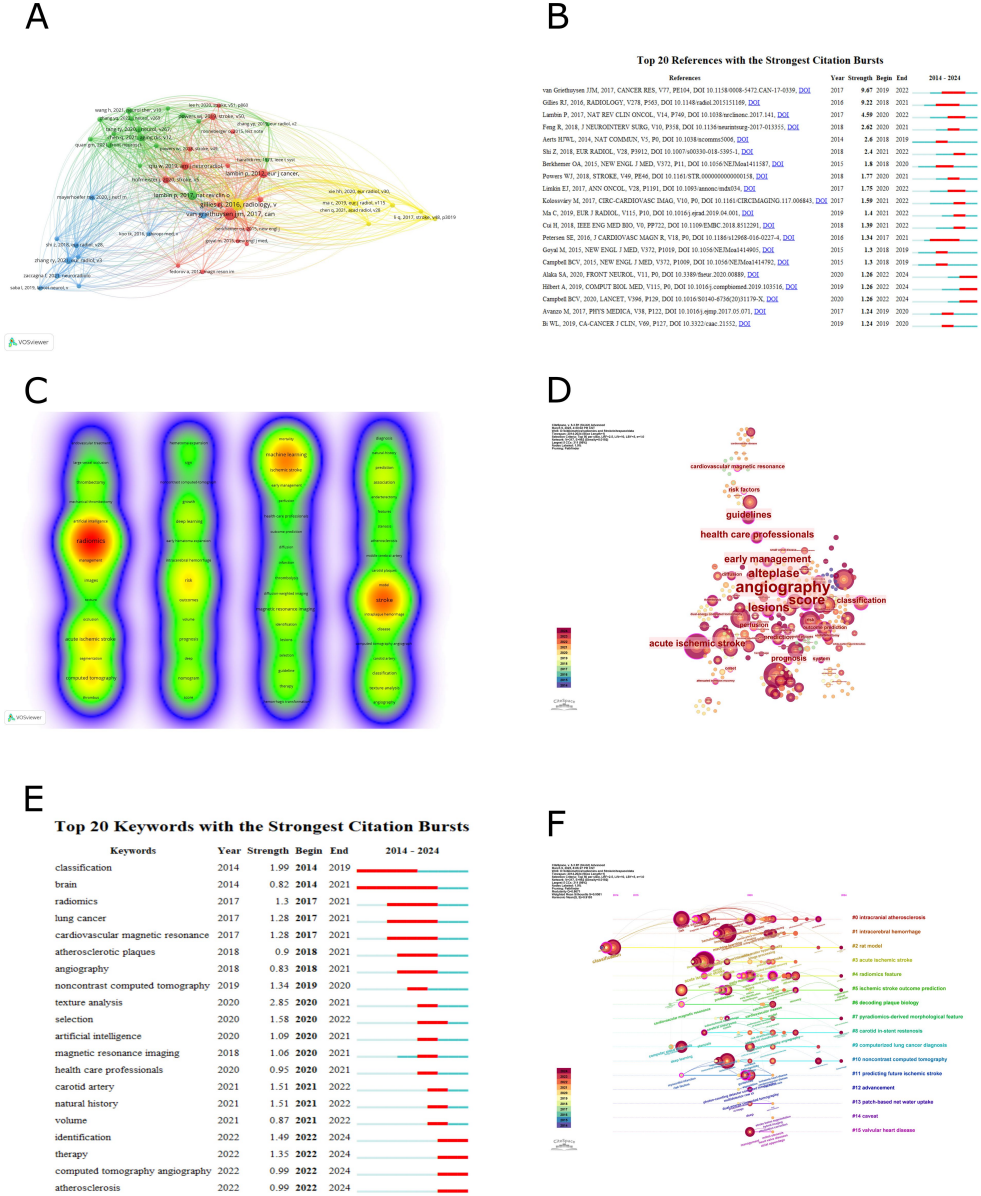
**(A)** Co-cited references analysis. **(B)** Sorted by strengths of burst. **(C)** Keyword co-occurrence analysis. **(D)** Keyword centrality analysis. **(E)** Sorted by beginning year of burst. **(F)** Timeline of keyword clustering analysis.

A co-citation analysis was conducted using VOSviewer to clarify the theoretical foundation and mainstream viewpoints in the field. Co-citation was selected for the type of analysis and cited references were selected for the unit of analysis, the threshold of the minimum number of citations in a cited reference was selected as 10. Among the 6,394 co-cited references, 51 papers had a co-citation count of 10 or more. Table 5 provides detailed information on the top 10 most co-cited papers. The papers “Radiomics: images are more than pictures, they are data” (2016) and “Computational radiomics system to decode the radiographic phenotype” (2017) were tied for the highest number of co-citations, both with 58. This was followed by “Radiomics: the bridge between medical imaging and personalized medicine” (2017) with 37 co-citations and “Radiomics: extracting more information from medical images using advanced feature analysis” (2012) with 35 co-citations. Figure 2A illustrates the visualization network of the 51 papers with 10 or more co-citations (4 clusters). Additionally, a burst analysis of co-cited literature was performed using CiteSpace, with a threshold parameter *γ* set to 0.5 while keeping other parameters at their default settings. The software identified 20 co-cited papers with burst characteristics, and these are presented in Figure 2B, ranked by the intensity of their bursts.

**Table 5:** Top 10 co-references in number of papers published in the field of radiomics for stroke.

	Title	Year	Frequency	Source/IF (2023)	First author	Article type	References
1	Radiomics: images are more than pictures, they are data	2016	58	Radiology/12.1/Q1	Gillies, R. J.	Special Report	(6)
2	Computational radiomics system to decode the radiographic phenotype	2017	58	Cancer Research/12.5/Q1	van Griethuysen, J. J. M.	Methodology Paper	(19)
3	Radiomics: the bridge between medical imaging and personalized medicine	2017	37	Nature Reviews Clinical Oncology/81.1/Q1	Lambin, P.	Review	(7)
4	Radiomics: extracting more information from medical images using advanced feature analysis	2012	35	European Journal of Cancer/7.6/Q1	Lambin, P.	Review	(15)
5	Radiomics-Based intracranial thrombus features on ct and cta predict recanalization with intravenous alteplase in patients with acute ischemic stroke	2019	28	American Journal of Neuroradiology/3.1/Q2	Qiu, W.	Original Research	(38)
6	Radiomics in stroke neuroimaging: techniques, applications, and challenges	2021	23	Aging and Disease/7/Q1	Chen, Q.	Review	(3)
7	Clot-based radiomics predict a mechanical thrombectomy strategy for successful recanalization in acute ischemic stroke	2020	23	Stroke/7.8/Q1	Hofmeister, J.	Original Research	(45)
8	Identification of high-risk carotid plaque with MRI-based radiomics and machine learning	2021	23	European Radiology/4.7/Q1	Zhang, R. Y.	Original Research	(46)
9	Guidelines for the early management of patients with acute ischemic stroke: 2019 update to the 2018 guidelines for the early management of acute ischemic stroke: a guideline for healthcare professionals from the American Heart Association/American Stroke Association	2019	22	Stroke/7.8/Q1	Powers, W. J.	Clinical Practice Guideline	(60)
10	Penumbra-based radiomics signature as prognostic biomarkers for thrombolysis of acute ischemic stroke patients: a multicenter cohort study	2020	20	Journal of Neurology/4.8/Q1	Tang, T. Y.	Original Research	(41)

### 3.7 Analysis of keyword co-occurrence

A comprehensive analysis of keyword co-occurrences was conducted using VOSviewer and CiteSpace to identify research hotspots in the field. Table 6 presents the top 26 high-frequency keywords from VOSviewer and the top 28 high-betweenness centrality keywords from CiteSpace. The VOSviewer data is sorted by keyword co-occurrence frequency, with the 10 most frequent keywords being “radiomics” (127), “stroke” (86), “machine learning” (53), “acute ischemic stroke” (48), “computed tomography” (45), “risk” (35), “ischemic stroke” (34), “magnetic resonance imaging” (26), “deep learning” (22), and “thrombectomy” (21). The CiteSpace data is sorted by betweenness centrality, and the 10 keywords with the highest centrality are “angiography” (0.59), “score” (0.51), “alteplase” (0.45), “lesions” (0.44), “early management” (0.37), “acute ischemic stroke” (0.35), “health care professionals” (0.35), “guidelines” (0.34), “classification” (0.29), and “prognosis” (0.27). Figure 2C illustrates the visualization of keyword co-occurrences in VOSviewer. A total of 817 keywords were retrieved, with 65 keywords having a co-occurrence frequency of at least 5 times. There are 940 total connections and a total co-occurrence strength of 2,532, resulting in 4 clusters. The closer the node color is to red, the higher the co-occurrence frequency. Additionally, keywords within the same vertical cluster have stronger associations. Figure 2D demonstrates the visualization of keyword centrality in CiteSpace, with a time slice selection of 1 and a top *N* selection of 50. A total of 317 keyword nodes are included, with 1,143 total connections. Due to the initial overwhelming size of the visualization network, a pruning process was applied. Nodes with a betweenness centrality exceeding 0.1 are outlined in purple in the figure, and larger node labels indicate higher betweenness centrality.

**Table 6:** Top 26 high-frequency keywords and top 28 betweenness centrality keywords in the field of radiomics for stroke.

VOSviewer				CiteSpace				
	Keyword	Frequency	Total co-occurrence strength		Keyword	Frequency	Centrality	Year
1	radiomics	127	568	1	angiography	6	0.59	2018
2	stroke	86	382	2	score	12	0.51	2020
3	machine learning	53	247	3	alteplase	2	0.45	2020
4	acute ischemic stroke	48	222	4	lesions	9	0.44	2021
5	computed tomography	45	235	5	early management	6	0.37	2020
6	risk	35	180	6	acute ischemic stroke	49	0.35	2018
7	ischemic stroke	34	150	7	health care professionals	10	0.35	2020
8	magnetic resonance imaging	26	124	8	guidelines	11	0.34	2020
9	deep learning	22	79	9	classification	16	0.29	2014
10	thrombectomy	21	95	10	prognosis	4	0.27	2019
11	nomogram	20	105	11	perfusion	4	0.24	2018
12	images	20	104	12	prediction	10	0.23	2019
13	prognosis	20	102	13	cardiovascular magnetic resonance	3	0.21	2017
14	outcomes	19	116	14	risk factors	2	0.21	2017
15	texture analysis	17	87	15	system	3	0.19	2019
16	association	17	86	16	diffusion	6	0.18	2022
17	classification	17	74	17	risk	34	0.17	2017
18	prediction	15	84	18	outcome prediction	18	0.17	2020
19	management	15	80	19	onset	2	0.16	2019
20	intracerebral hemorrhage	14	86	20	endarterectomy	8	0.12	2021
21	hematoma expansion	13	83	21	stenosis	6	0.12	2018
22	guideline	13	69	22	carotid plaques	4	0.12	2023
23	thrombolysis	12	67	23	recovery	4	0.12	2022
24	score	12	66	24	dual-energy computed tomography	3	0.12	2020
25	disease	12	61	25	thrombolysis	10	0.1	2019
26	atherosclerosis	12	59	26	attenuated inversion recovery	3	0.1	2021
				27	small vessel disease	3	0.1	2019
				28	hemorrhage	2	0.1	2024

### 3.8 Analysis of keyword burst

Using CiteSpace for keyword burst analysis, select top 50 levels of most cited or occurred items from each slice, the threshold parameter *γ* was set to 0.3 while keeping other parameters at their default values. The software identified 20 keywords with burst characteristics, arranged by the starting year of the burst and illustrated in Figure 2E. The popular keywords before 2021, as indicated by the software, were “texture analysis,” “classification,” “selection,” “carotid artery,” and “natural history.” Their burst strengths were 2.85, 1.99, 1.58, 1.51, and 1.51, respectively. The popular keywords after 2022 were “identification,” “therapy,” “computed tomography angiography,” and “atherosclerosis.” Their corresponding burst strengths were 1.49, 1.35, 0.99, and 0.99.

### 3.9 Analysis of keyword clustering

We conducted a keyword clustering analysis using the CiteSpace software, select top 50 levels of most cited or occurred items from each slice. Specifically, we employed the timeline visualization feature in CiteSpace to cluster the keywords. Utilizing the log-likelihood ratio (LLR) algorithm, we categorized the keywords into distinct thematic groups. The software generated 15 clusters, with each cluster having a silhouette value of at least 0.88, indicating high credibility of the clustering modules. Table 7 provides detailed information on the top 10 clusters, while the visual timeline is presented in Figure 2F. The modularity (*Q*) of 0.8671 and the weighted average silhouette (*S*) of 0.9581 demonstrate that the keyword clusters derived from the data possess significant statistical meaning and high reliability. Within the primary cluster “#0 intracranial atherosclerosis,” the betweenness centrality of “angiography,” “outcome prediction,” “endarterectomy,” and “carotid plaques” all exceed 0.1. The top 10 most recently emerging keywords across all clusters are “prediction model,” “systematic review,” “veins,” “futile recanalization,” “carotid ultrasound,” “hemorrhage,” “carotid plaques,” “atrial fibrillation,” “health,” and “mechanical thrombectomy.

**Table 7:** Top 10 keyword clusters.

Clusters	Size	Silhouette	Keywords
#0	38	0.893	intracranial atherosclerosis; high-risk carotid plaque; radiomic approach; initial experience; high-risk plaque feature
#1	26	0.996	intracerebral hemorrhage; hematoma expansion; spontaneous intracerebral hemorrhage; hypertensive intraparenchymal hematoma; predicting hematoma expansion
#2	26	0.983	rat model; microvascular MRI; unsupervised clustering yield; 3D high-resolution magnetic resonance; high-risk intracranial plaque
#3	25	0.977	acute ischemic stroke; stroke management; clinical application; hyperacute stroke; relative radiomic pattern
#4	25	0.944	radiomics feature; penumbra-based radiomics signature; prognostic biomarker; multicenter cohort study; new chance
#5	22	0.887	ischemic stroke outcome prediction; whole brain; infarct lesion; ischaemic stroke lesion; texture analysis
#6	20	1	decoding plaque biology; noninvasive phenotyping; virtual transcriptomics; UK Biobank; fish consumption
#7	18	0.956	pyradiomics-derived morphological feature; aneurysm stability; machine learning model; techniques application; radiomics difference
#8	18	0.964	carotid in-stent restenosis; computed tomography angiography carotid; plaque-based radiomics; diagnostic tool; CT radiomics feature
#9	16	0.962	computerized lung cancer diagnosis; using multichannel ROI; automatic feature learning; deep structured algorithm; artificial intelligence integration

## 4 Discussion

To the best of our knowledge, this study represents the first bibliometric analysis examining research trends in radiomics within the field of ischemic stroke. The search period was set from January 1, 2004, to December 31, 2024, resulting in the initial retrieval of 211 studies from the WoSCC database. Two researchers independently reviewed all articles by examining their titles, abstracts, and full texts. Any disagreements were fully discussed until a consensus was reached. Ultimately, a total of 203 papers were included in the study. We utilized VOSviewer 1.6.18 and CiteSpace 6.3.R1 to conduct a bibliometric analysis on the selected articles. The analysis encompassed various aspects, including the countries of origin, publishing institutions, authors/co-cited authors, and co-citations, aiming to summarize the current research landscape in this domain. Additionally, we performed keyword co-occurrence analysis, cluster analysis, and keyword emergence analysis to identify trends and potential hotspots in radiomics research related to ischemic stroke.

### 4.1 General description

The analysis of publication trends presented in Figure 1A untangles the development of radiomics in the field of ischemic stroke. Based on the trend line formula in the graph, it is evident that the number of publications in this domain exhibits a linear growth pattern. The first piece of literature within the search scope was published in 2014 by Coquery et al. (10) in the Journal of Cerebral Blood Flow & Metabolism, titled “Microvascular MRI and unsupervised clustering yields histology-resembling images in two rat models of glioma”. This groundbreaking glioma study proposes a multiparametric MRI integration model (incorporating ADC, vascular permeability, blood volume, blood flow, and oxygenation/metabolic indices), which overcomes the limitations of single-parameter analysis through an unsupervised clustering framework. Although primarily applied to tumor microenvironment heterogeneity analysis, it establishes a quantitative analytical paradigm for microcirculatory dysfunction in ischemic stroke radiomics. The computational architecture provides a methodological foundation for subsequent lesion heterogeneity quantification studies, establishes a validation template for MRI-histopathological feature mapping, and implements a systematic pipeline for biomarker discovery in cerebrovascular pathological mechanisms. This methodological transfer significantly advances ischemic stroke radiomics from a phenotype-observation to a mechanism-driven paradigm. Since then, radiomics research in the field of ischemic stroke has gradually garnered attention from scholars, becoming a popular research direction in recent years. Notably, relevant papers published in just 2023 and 2024 account for more than 50% of the total publications.

Figure 1B and Table 1 present a visual map and specific data on global publications and collaborations among countries. In terms of the number of publications, China leads with 147 published studies, accounting for 72% of the total research output, followed by the United States with 37 publications in second place. However, from the perspective of international cooperation, the United States ranks first with a total cooperation strength of 77, followed by Canada and the UK. The lines connecting nodes in the figure represent the strength of cooperation, and it can be observed that the thickest line connects China and the United States, indicating the strongest connection between the two countries in this field. It’s worth noting that despite China’s leading position in publication quantity, its cooperation strength ranks only 17th, suggesting that Chinese research is primarily focused on domestic collaborations rather than international ones.

Figure 1C and Table 2 present the visualization network and detailed data of the publication quantity and collaboration among global research institutions. Among the top 10 research institutions, General Electric Healthcare from the United States ranks first with 13 studies, while the remaining institutions are all located in China, aligning with China’s leading position in terms of publication volume. In Figure 1C, we observe the formation of six clusters of different sizes among various research institutions. The nodes with a higher number of publications belong to different clusters, indicating that the research in this field has established numerous and complex inter-institutional collaborations. These collaborations encompass both small-scale research groups and cross-cluster cooperative connections.

Table 3 presents the specific details of publication volume and collaboration relationships among global authors. The top 10 authors in terms of publication volume are all from China, which correlates with China’s position as the global leader in overall publications. Two professors from Northeastern University, Guo, Yingwei, with an H-index of 8, and Kang, Yan, with an H-index of 6, share the first position with 8 research papers each. Guo, Yingwei, and Kang, Yan, have closely collaborated on research primarily focused on enhancing the accuracy of ischemic stroke diagnosis and prognosis prediction using radiomics and artificial intelligence techniques (11–13). Figure 1D visualizes the collaboration between authors, revealing dense cooperation within clusters and less pronounced collaboration between clusters. This indicates that research in this field is primarily explored independently by various research groups, and large-scale cross-cluster collaboration patterns have not yet emerged.

Additionally, Table 3 showcases the top 15 co-cited authors, with Lambin, P. (73), Saba, L. (61), Gillies, R. J. (59), and van Griethuysen, J. J. M. (58) leading the list. Figure 1E illustrates the visualization of co-cited authors, highlighting Lambin, P., Gillies, R. J., van Griethuysen, J. J. M., and others as larger and centrally located nodes. This suggests their pivotal role in the co-citation network, with broad co-citation relationships spanning multiple clusters, indicating that their research likely forms the theoretical foundation or key perspectives in the field. Lambin, P., a professor of radiation oncology at Maastricht University in the Netherlands, has an H-index of 128. He has received ERC advanced and twice ERC PoC grants in 2016, 2017, and 2020, and is a pioneer in translational research focusing in tumor hypoxia, genetically modified bacteria for cancer treatment, and decision support systems. His core research areas include the development of radiomics methodologies (7, 14, 15), AI-driven prognostic models for tumors (16), and the discovery of biomarkers for radiotherapy response (17). Gillies, R. J., who passed away on June 7, 2022, served as the chair of the Department of Cancer Physiology at the Moffitt Cancer Center and was a member of the AACR. He is remembered for his outstanding contributions to cancer research, with an H-index of 129. His work focused on cancer metabolism (18) and radiomics (6), and he made groundbreaking contributions to interdisciplinary cancer research, particularly in integrating physiology and imaging. van Griethuysen, J. J. M., a scholar from Maastricht University in the Netherlands with an H-index of 13, specializes in the automated processing of medical images and the application of deep learning models (19). Notably, Lambin, P., Gillies, R. J., and van Griethuysen, J. J. M., have collectively contributed to the Image Biomarker Standardization Initiative (20). The relatively independent purple cluster centered around Saba, L. suggests that his research field may be more concentrated on a single theme, with co-citations focused within a narrower scope. Saba, L. is a professor of radiology at the University of Cagliari in Italy and the director of the radiology department at the university hospital. He serves as an editorial board member for over 10 SCI journals, including the American Journal of Neuroradiology, and has an H-index of 78. His primary research areas include carotid plaque radiomics (21, 22), novel neurovascular imaging techniques (23), and AI-assisted diagnosis (24).

Table 4 describes the specific details of the journals that published the articles. In terms of the number of publications, *Frontiers in Neurology* (26) is the journal with the highest number of published studies, followed by *Frontiers in Neuroscience* (14) and *European Radiology* (12). Regarding the journal’s level, the JCR partitions of the top 10 journals publishing research in this field are all no lower than Q2, indicating that the research in this area is sufficiently recognized by high-level journals. Figure 1F illustrates the visualization of the publishing journals. There are 36 journals that have published 2 or more articles, and the figure shows the largest subset (7 clusters) of 35 journals with citation relationships. Although several journals with a higher number of publications are located in different clusters, they have close citation connections, indicating both mutual confirmation of viewpoints and differences in research foci among the studies published by various journals.

### 4.2 Hot spots and frontiers

This study employed VOSviewer and CiteSpace to analyze the co-citation of references and the co-occurrence of keywords, aiming to identify research trends and changes in hotspots within the field. Table 5 presents detailed information on the top 10 most co-cited references in this domain (co-citation of references refers to a situation where one reference is cited by two studies simultaneously, counted as one co-citation, indicating that the two studies share similar viewpoints or theoretical foundations). The most frequently co-cited reference is a special report published by Gillies et al. (6) in Radiology in 2016. This study innovatively proposed a radiomics analysis framework based on the fusion of quantitative features from multimodal medical imaging and genomic data. It systematically validated the unique value of radiomics in characterizing tumor heterogeneity, predicting prognosis, and evaluating treatment response. This approach of transforming images into high-dimensional data provides clinicians with a novel research paradigm for pursuing precision medicine. Notably, it offers valuable research recommendations for subsequent studies, including “curation of high-quality datasets,” “health informatics,” and “data sharing,” which are anticipated to emerge as potential research hotspots in future investigations. Due to its methodological significance, this study has become the most co-cited radiomics literature in the field of ischemic stroke research. Another notable reference is a methodology paper published by van Griethuysen et al. (19) in Cancer Research in 2017. This study has innovatively developed the open-source PyRadiomics platform, which standardizes the radiomic feature extraction workflow through image loading and preprocessing, multi-step feature screening, and comprehensive feature computation (including statistical and texture feature extraction, shape descriptor quantification, as well as 2D slice-based and 3D volume-based feature calculations). The platform incorporates a multimodal imaging compatibility design, systematically addressing critical industry challenges such as algorithmic inconsistency and non-comparable results in radiomics analysis. It provides essential infrastructure for reproducible quantitative research in radiomics. Lambin’s et al. (7) review published in Nature Reviews Clinical Oncology in 2017 is also worth mentioning. Building upon previous studies by Gillies et al. (6) and van Griethuysen et al. (19) and others, this work systematically examines the limitations, challenges, and opportunities of radiomics in enhancing clinical decision support systems for personalized precision medicine, with particular emphasis on the methodological rigor in developing and validating radiomics predictive models (including standardized nomenclature, algorithmic variations, software implementation differences, and other methodological considerations). The authors innovatively propose a radiomics technical framework comprising 16 core components, termed the Radiomics Quality Score (RQS). Through systematic construction of a comprehensive technical pathway encompassing: (1) “Image feature extraction” (requiring clinicians to explicitly define data requirements; addressing data heterogeneity challenges via phantom studies, multi-timepoint imaging/test-retest reliability data, and multiple segmentation approaches). (2) “Model validation” [discriminative performance characterized by receiver operating characteristic (ROC) curves or area under the curve (AUC); calibration assessed through calibration plots and integrated calibration measures (calibration-in-the-large/slope); with emphasis on both internal and external validation]. (3) “Clinical translation” (establishing certified radiogenomics centers and conducting cost-comparative analyses of quality-adjusted life years with/without radiomics integration), this study achieves, for the first time, multidimensional correlation analysis between quantitative imaging features and tumor heterogeneity, treatment response, and genomic characteristics. It establishes a reproducible methodological paradigm for image-driven precision diagnostics and treatment. Notably, the authors demonstrate that radiomics analysis is not confined to radiotherapy applications but can be extended to any medical imaging data generated in clinical practice. Figure 2A illustrates the visualization of co-cited references in this field. Among the 6,394 co-cited references, 51 (forming 4 clusters) have been co-cited at least 10 times. The proximity of multiple larger nodes belonging to different clusters indicates both distinct research foci and academic crossover among these co-cited references. Figure 2B shows the visualization of the emergence of co-cited references. A higher emergence intensity signifies a higher citation frequency of the reference within a specific time frame, and the year represents the period of surge in citation frequency. According to the figure, recent research hotspots have shifted from previous methodological studies to clinical application studies. This includes a multicenter cohort study published by Alaka et al. (25) in Frontiers in Neurology in 2020, which, for the first time, confirmed the equivalent efficacy of machine learning models based on multimodal imaging parameters (such as multiphase CT angiography) and logistic regression in predicting functional prognosis after acute ischemic stroke. This provided crucial methodological evidence for constructing a precise prognosis evaluation system driven by radiomics in stroke. A study by Hilbert et al. (26), published in Computers in Biology and Medicine in 2019, developed a data-efficient deep learning model based on CT angiography images that can directly predict functional recovery after endovascular treatment in patients with acute ischemic stroke. This offered a new paradigm for radiomics in stroke prognosis evaluation that does not rely on traditional biomarkers. Lastly, a review by Campbell and Khatri (1), published in Lancet in 2020, established an innovative application paradigm for CT/MRI radiomics in acute stroke treatment by defining the rescue time window of the penumbra and a treatment pathway based on the ASPECTS score (Alberta Stroke Program Early CT Score).

A 2022 retrospective study developed and validated a predictive model for hemorrhagic transformation (HT) risk based on radiomic features derived from non-contrast-enhanced CT scans of acute ischemic stroke patients (27). Through analysis of infarction zone characteristics in 118 AIS cases, five key radiomic features were selected to construct a Rad-score system. The model demonstrated AUC values of 0.845 and 0.750 in the training and validation cohorts, respectively, effectively evaluating HT risk across different treatment modalities (including intravenous thrombolysis and mechanical thrombectomy) and infarct volumes. These findings provide crucial reference for clinical decision-making. A 2024 retrospective study proposed a novel approach utilizing perfusion radiomic features to assess neurological impairment in AIS (28). Among the original perfusion parameters, significant differences were observed between patients with favorable versus poor neurological outcomes regarding cerebral blood flow and mean transit time in ischemic regions. Comparative experiments demonstrated that radiomic features from ischemic regions (achieving an AUC of 0.923 with Random Forest modeling) significantly outperformed conventional perfusion parameters (AUC 0.868). Radiomic features from hypoxic zones showed inferior performance (AUC = 0.769), underperforming even their original parameters (AUC = 0.876). The combination of features or parameters from multiple regions failed to surpass the predictive value of single ischemic region features, showing only marginal improvement for infarct/hypoxic zones. These findings confirm that ischemic regions alone provide the most clinically relevant information, offering a more objective tool for neurological impairment assessment. A 2024 cohort study employed machine learning models to evaluate the predictive value of radiomic features from admission head CT scans—specifically intracerebral hemorrhage (ICH) and perihematomal edema (PHE)—for 3-month poor functional outcomes (modified Rankin Scale scores 4–6) (29). The results demonstrated that incorporating PHE features into ICH radiomics significantly improved individual-level risk assessment [with integrated discrimination improvement (IDI) and net reclassification improvement (NRI) showing *p* < 0.001], though the overall prognostic accuracy [area under the curve (AUC)] did not show significant enhancement (0.74 vs. 0.71, *p* = 0.157). The combined model integrating clinical variables with radiomic features (AUC = 0.85) significantly outperformed traditional ICH scoring systems, providing immediate and quantitative risk stratification evidence to guide interventions such as hematoma evacuation. A 2024 review summarized studies on post-stroke cognitive impairment (encompassing both ischemic and hemorrhagic stroke) utilizing lesion-symptom mapping techniques (30). This research localized key brain regions (e.g., angular gyrus and basal ganglia) associated with executive dysfunction and language deficits, supporting early intervention strategies. The review concurrently identified methodological challenges, including data heterogeneity (e.g., inconsistent definitions of post-stroke cognitive impairment) and limited generalizability of computational models due to small sample sizes. These issues necessitate multicenter data sharing and standardized feature extraction protocols for resolution.

In a retrospective study analyzing futile recanalization in patients with anterior circulation AIS subjected to endovascular thrombectomy (EVT), 2016 radiomic features were extracted from non-contrast computed tomography (NCCT) images. Nine optimal features were ultimately selected to construct a radiomic model (31). Admission National Institutes of Health Stroke Scale (NIHSS) score, hemorrhagic transformation, neutrophil-to-lymphocyte ratio (NLR), and admission blood glucose were identified as independent predictive factors. The radiomic-clinical nomogram model demonstrated AUC values of 0.860 and 0.775 in the training and validation cohorts, respectively. This integrated model, combining radiomic and clinical features, outperformed standalone radiomic or clinical models, demonstrating its potential for early prediction of AIS patient outcomes and assisting clinicians in formulating personalized treatment strategies. Additionally, inflammatory markers and blood glucose levels were found to play significant roles in predicting futile recanalization. The study validated the feasibility of NCCT in prognostic assessment for stroke patients. Despite limitations such as its single-center design, the model exhibits promising clinical applicability. A multicenter study developed a clinical-radiomics model based on NCCT to predict HT risk following intravenous thrombolysis (IVT) in AIS patients using machine learning (32). The study enrolled 517 patients from seven hospitals. Through recursive feature elimination and extreme gradient boosting, 12 profound radiomic features were selected for model construction, leading to the development of a clinical model, a radiomics model, and a combined clinical-radiomics model. The model demonstrated stable performance in multicenter validation, exhibiting strong generalizability and completing HT risk assessment for new patients within 1 min, highlighting its clinical utility. Results showed that the combined model achieved optimal performance in both internal and external validation cohorts, with AUC values of 0.950 and 0.942, respectively. These findings confirm that the model provides a reliable tool for assessing HT risk in stroke patients after IVT.

In a systematic review evaluating the utility of machine learning for predicting the time of symptom onset in ischemic stroke patients (33), 13 studies comprising a total of 55 models were included. The most frequently employed models were logistic regression, support vector machine (SVM), boosting, and random forest. Subgroup analysis demonstrated the highest predictive accuracy for symptom onset within 4.5 h, with logistic regression exhibiting optimal performance among the models. In a systematic review aimed at predicting HT following thrombolytic therapy in posterior circulation ischemic stroke (34), researchers analyzed 12 global clinical studies encompassing 18,007 AIS patients subjected to thrombolysis, with a mean age range of 64–69 years. The extreme gradient boosting (XGBoost) and artificial neural network (ANN) models demonstrated optimal performance, achieving area under the curve (AUC) values of 0.953 and 0.942 in internal validation, respectively, while the pooled AUC for external validation reached 0.80. Core predictors of HT included age, blood glucose levels, NIHSS scores, systolic/diastolic blood pressure, and radiomic imaging features (e.g., vascular health indicators). Models integrating clinical data with radiomic characteristics significantly enhanced predictive accuracy, attaining peak sensitivity of 0.90 and specificity of 0.99. Study limitations primarily stemmed from methodological heterogeneity, particularly evident in inconsistent HT definitions, limited external validation (conducted in only 50% of studies), and divergent missing-data handling strategies (e.g., multiple imputation vs. case exclusion). Funnel plot analysis suggested potential publication bias, underscoring the need for improved model transparency and multicenter validation to ensure result reliability.

Keywords as a high-level summary of research content can help us identify current research foci and potential hotspots. Table 6 presents the keyword recognition of the retrieved literature by two software programs. In VOSviewer besides the search keywords “radiomics” and “stroke” used in this study other high-frequency keywords include “machine learning” “acute ischemic stroke” “computed tomography” “risk” and “ischemic stroke.” Figure 2C illustrates the visualization of keyword co-occurrence in VOSviewer. Among 817 keywords there are 65 keywords (in 4 clusters) with a co-occurrence frequency of at least 5 times resulting in a total of 940 connections and a total co-occurrence strength of 2,532. The total co-occurrence strength refers to the sum of the connection strengths between a keyword and all other keywords in the keyword co-occurrence network. Keywords with higher total connection strength typically indicate stronger co-occurrence relationships with more keywords suggesting they may occupy a central position in a research field. By comprehensively analyzing the VOSviewer keywords in Table 6 and the clustering in Figure 2C we found a close research relationship between “radiomics” and “acute ischemic stroke” “computed tomography.” Radiomics utilizes the clinical advantages of computed tomography (CT) such as fast imaging and safety profile along with its high-throughput feature extraction capability to address key challenges in ischemic stroke including early lesion detection and treatment outcome prediction (3, 27). There is also a strong research connection between “machine learning” and “ischemic stroke” “magnetic resonance imaging.” Machine learning significantly improves diagnostic and therapeutic accuracy in ischemic stroke MRI analysis through automatic lesion segmentation (using the U-Net architecture) and prognostic prediction models (utilizing the XGBoost algorithm) (35–37). Figure 2D displays the visualization of keyword nodes in CiteSpace showing 317 keyword nodes with 1,143 total connections. Nodes with labels have a betweenness centrality of no less than 0.1. Betweenness centrality is a metric in bibliometrics that measures a node’s bridging role in a citation network. Nodes with a betweenness centrality exceeding 0.1 are often considered key nodes connecting different research fields serving as bridges for interdisciplinary knowledge flow. Table 6 highlights nodes with high betweenness centrality such as “angiography” (0.59) (38, 39) “score” (0.51) (40) “alteplase” (0.45) (41) “lesions” (0.44) (42) and “early management” (0.37) (8, 43). These keywords may represent current or future interests in cross-disciplinary research. Figure 2E shows the visualization of keyword emergence in CiteSpace. Popular keywords before 2021 include “texture analysis” (3) “classification” (44) “selection” (45) “carotid artery” (46) and “natural history” (47). After 2022 popular keywords are “identification” (43) “therapy” (48) “computed tomography angiography” (49) and “atherosclerosis” (50). Table 7 details the top 10 keyword clusters all with Silhouette values of at least 0.88 indicating high clustering accuracy. Figure 2F presents a timeline of keyword clusters revealing emerging keywords such as “prediction model” (51) “systematic review” (8) “veins” (52) “futile recanalization” (53) “carotid ultrasound” (54) “hemorrhage” (55) “carotid plaques” (56) “atrial fibrillation” (57) and “mechanical thrombectomy” (58).

### 4.3 Strategies to overcome translational barriers in clinical practice

Our study proposes potential solutions to address existing translational challenges in clinical applications. Regarding methodological heterogeneity, the following should be provided as Supplementary material: imaging acquisition protocols, scan data for analysis, volume of interest (VOI) segmentation results, detailed feature extraction procedures (including calculation formulas), and modeling methods (with open-source code recommended). Only through such meticulous disclosure can reproducibility and replicability be effectively validated. In terms of data preservation, we recommend exploring an interdisciplinary precision medicine consensus framework bridging research and clinical practice to establish a shared database applicable to real-world health studies. Leveraging the “4V” characteristics of databases—“Volume” (data scale), “Variety” (data diversity), “Velocity” (data timeliness), and “Veracity” (data authenticity)—new research approaches can be developed to mitigate current data-sharing barriers (7). Notably, the “CancerLinQ” initiative (59), proposed by the American Society of Clinical Oncology (ASCO), has advanced this objective through data centralization strategies. To address cultural and linguistic heterogeneity, semantically interoperable datasets should be established as unified reference standards across institutional sites. This approach facilitates standardized data management while ensuring cross-institutional compatibility.

### 4.4 Future prospects

Future research must focus on addressing the following three critical directions: (1) Multicenter dynamic data integration: A decentralized validation platform based on federated learning (FL) should be established, with dynamic image acquisition protocols developed in accordance with the American College of Radiology (ACR) Imaging Biomarker Certification Standards. By modeling the evolutionary patterns of dynamic features during treatment, the accuracy of image-guided interventional therapy can be significantly improved. (2) Clinically applicable explainable artificial intelligence: Attention heatmap tools incorporating gradient-weighted class activation mapping (Grad-CAM++) should be developed, integrating automated region-of-interest (ROI) segmentation and historical image comparison functions. Additionally, a multimodal decision tree combining the National Institutes of Health Stroke Scale (NIHSS) scores with coagulation parameters should be constructed to translate high-performance models [e.g., modified Alberta Stroke Program Early CT Score (ASPECTS), with an area under the curve (AUC) of 0.89] into reliable clinical tools for prognostic assessment and treatment planning. (3) Translational validation of dynamic systems: key efforts should include: (i) Acute phase (<6 h): prospective validation of thrombolysis/thrombectomy decision support systems in clinical trials (e.g., MR CLEAN study). (ii) Chronic phase (90 days): establishment of a dynamic monitoring system linking the modified Rankin Scale (mRS) with radiomics to enable 24-h risk-level updates. Ultimately, individualized treatment response assessment will advance the development of precision medicine.

## 5 Conclusion

Radiomics is transforming ischemic stroke research paradigms. This study maps the field’s evolution: from early feature extraction standardization, to mid-phase machine learning prognostic models, culminating in multimodal imaging-treatment system integration. While Chinese scholars lead in publication volume, limited international collaboration and clinical translation remain bottlenecks. CTA-based radiomics show clinical value in acute ischemic stroke vascular assessment but require validation for hemorrhagic stroke and long-term prognosis. Future priorities must focus on: (1) Multicenter integration: FL-based validation platforms with ACR-compliant dynamic protocols to enhance image-guided therapy accuracy. (2) Explainable AI: Grad-CAM++ tools with automated ROI segmentation combined with NIHSS/coagulation decision trees to operationalize models like modified ASPECTS (AUC = 0.89). (3) Translational validation: (i) prospective thrombolysis/thrombectomy validation (<6 h; e.g., MR CLEAN). (ii) mRS-radiomics linked chronic monitoring systems enabling 24-h risk updates. Only through integrated technological innovation can radiomics advance from scientific exploration to routine clinical implementation.

## 6 Limitations

The limitations of this study are as follows: (1) Regarding data sources, although the Web of Science (WoS) database is authoritative, its insufficient coverage of Asian regional journals may result in the underestimation of research contributions from non-English speaking countries outside China. (2) Methodologically, bibliometric tools such as VOSviewer have limited capabilities in disambiguating author/institution names, which may affect some collaboration network analyses due to data cleaning errors. (3) In terms of timeliness, key technological advancements published after December 2024, such as Transformer-based image segmentation algorithms, were not included, potentially impacting the assessment of cutting-edge trends.

## Data Availability

The raw data supporting the conclusions of this article will be made available by the authors, without undue reservation.
