# Supplementary material for: Radiomics and ischemic stroke research: bibliometric insights and visual trends (2004–2024)
Source: Front Neurol. 2025 Aug 28;16:1606388. doi: 10.3389/fneur.2025.1606388 (PMC12422918; doi:10.3389/fneur.2025.1606388)
Supplement: Supplementary file 1 [file Data_Sheet_1.pdf]

Supplementary Table

Table S1

search strategy by 2025-02-08

Serial numbers	Search modes	results
#1	TS=((Stroke) OR (Strokes) OR (Cerebrovascular Accident) OR (Cerebrovascular Accidents) OR (Cerebral Stroke) OR (Cerebral Strokes) OR (Stroke, Cerebral) OR (Strokes, Cerebral) OR (Cerebrovascular Apoplexy) OR (Apoplexy, Cerebrovascular) OR (Vascular Accident, Brain) OR (Brain Vascular Accident) OR (Brain Vascular Accidents) OR (Vascular Accidents, Brain) OR (Cerebrovascular Stroke) OR (Cerebrovascular Strokes) OR (Stroke, Cerebrovascular) OR (Strokes, Cerebrovascular) OR (Apoplexy) OR (CVA (Cerebrovascular Accident)) OR (CVAs (Cerebrovascular Accident)) OR (Stroke, Acute) OR (Acute Stroke) OR (Acute Strokes) OR (Strokes, Acute) OR (Cerebrovascular Accident, Acute) OR (Acute Cerebrovascular Accident) OR (Acute Cerebrovascular Accidents) OR (Cerebrovascular Accidents, Acute) OR (basilar artery occlusion) OR (brainstem infarction) OR (ischemic stroke) OR (ischaemic stroke) OR (cerebral infarction) OR (brain infarction) OR (cerebral ischemia) OR (brain ischemia) OR (thrombotic stroke) OR (embolic stroke) OR (thromboembolic stroke) OR (cerebral thromboembolism) OR (acute ischemic cerebrovascular syndrome))	851,248
#2	TS=((Radiomic) OR (Radiomics))	16,074
#3	#1 AND #2	250
#4	#3 AND DOP=(2004-01-01/2024-12-31) AND LA=(English) AND DT=(Article OR Review)	227
