# Supplementary figures and images for: Radiomics and ischemic stroke research: bibliometric insights and visual trends (2004–2024)
Source: Front Neurol. 2025 Aug 28;16:1606388. doi: 10.3389/fneur.2025.1606388 (PMC12422918; doi:10.3389/fneur.2025.1606388)

Supplementary Figure  
Figure S1

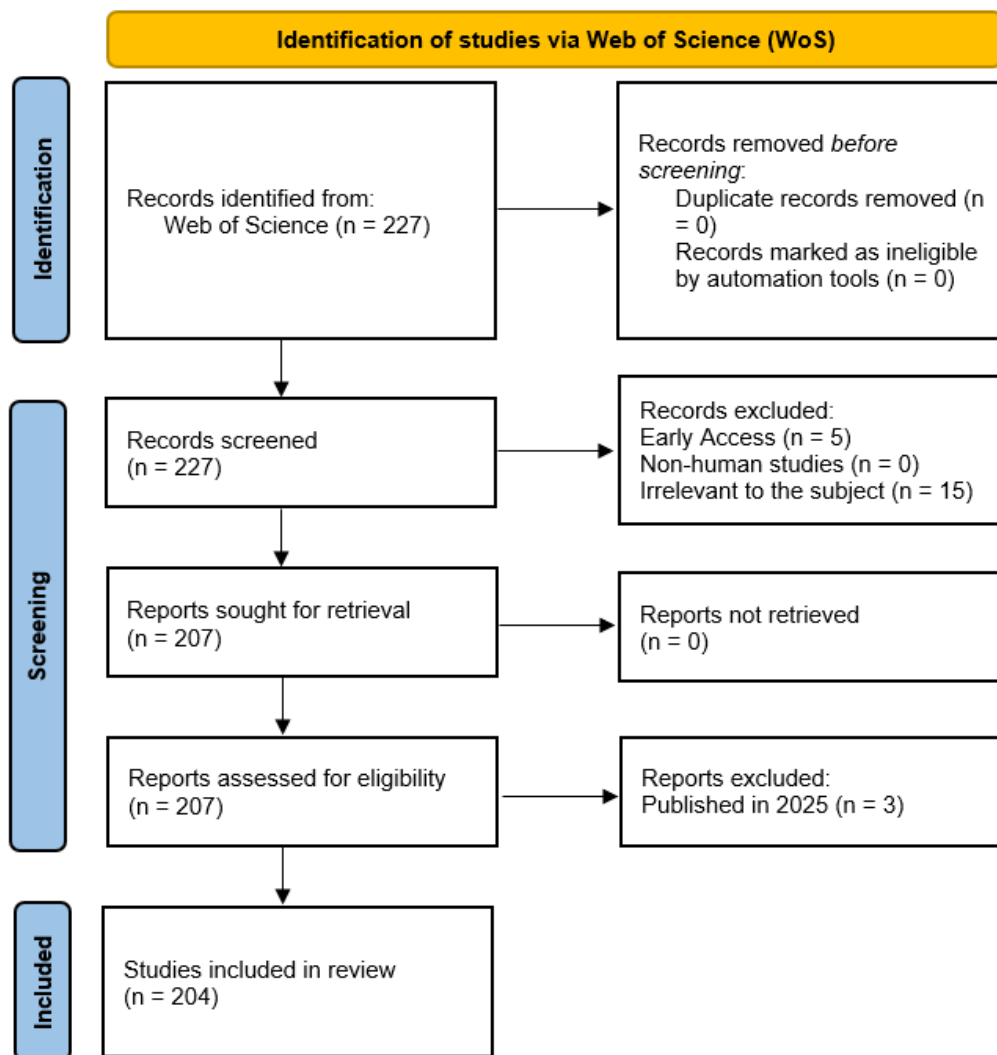

Supplement: Supplementary file 2 [file Data_Sheet_2.pdf]
